# First person – Andrea Raposo López-Pastor

**DOI:** 10.1242/dmm.038976

**Published:** 2019-02-07

## Abstract

First Person is a series of interviews with the first authors of a selection of papers published in Disease Models & Mechanisms, helping early-career researchers promote themselves alongside their papers. Andrea Raposo López-Pastor is first author on ‘Liver-specific insulin receptor isoform A expression enhances hepatic glucose uptake and ameliorates liver steatosis in a mouse model of diet-induced obesity’, published in DMM. Andrea is a PhD student in the lab of Manuel Benito at Complutense University of Madrid, Madrid, Spain, investigating mechanisms implicated in progression of fatty liver and its relation with other metabolic pathologies.


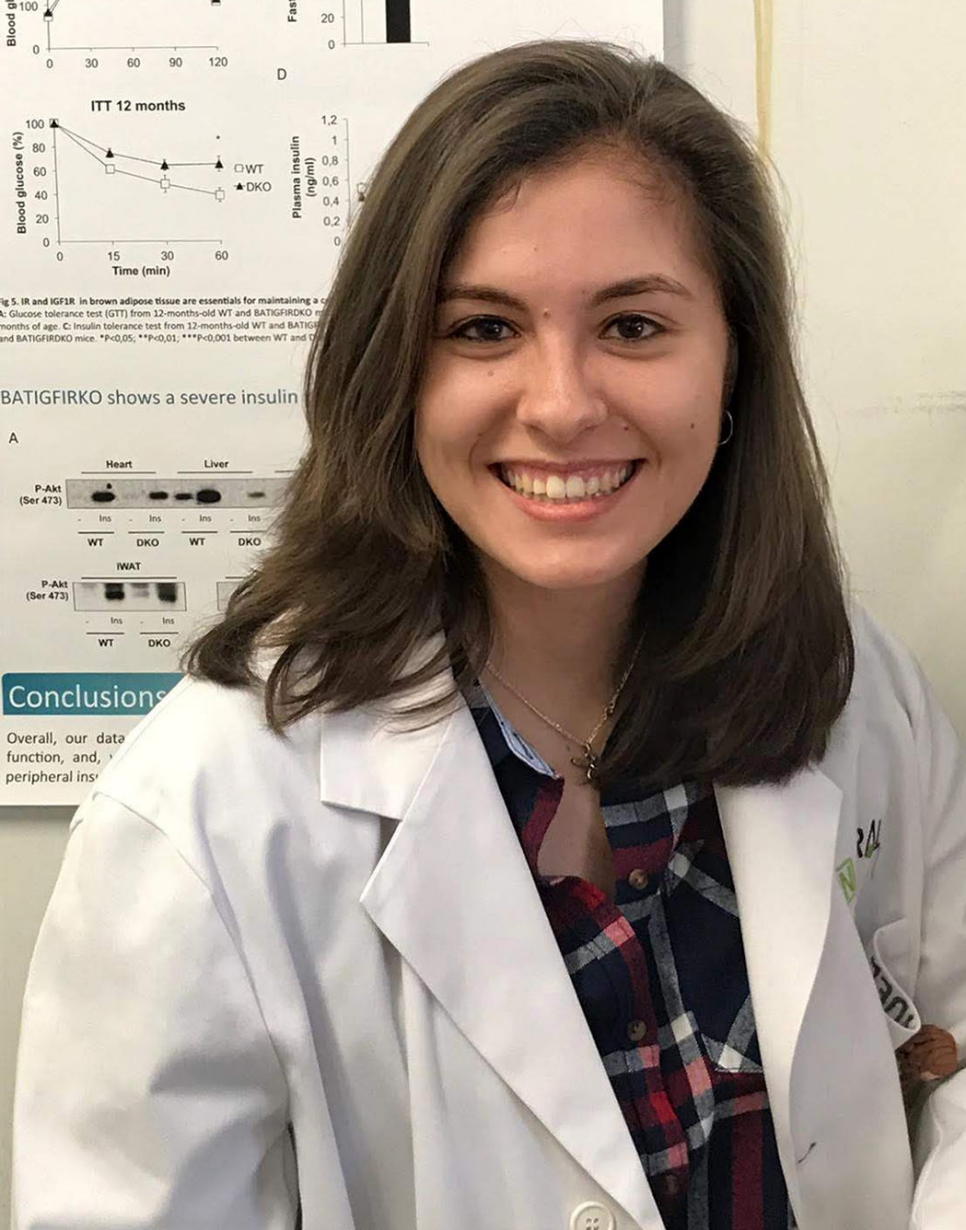


**Andrea Raposo López-Pastor**

**How would you explain the main findings of your paper to non-scientific family and friends?**

Nowadays, due to the availability of hypercaloric food and sedentarism, obesity is considered a great health problem worldwide. Among the main complications associated with obesity are the loss of insulin action and altered glucose and lipid metabolism within the liver. As has been previously reported, mice without hepatic insulin function were approached by gene therapy using two human forms of insulin receptor. These findings showed that insulin receptor type A (IRA) improved some signs of diabetic disease compared to type B (IRB). But, as obesity is the main cause of diabetes, we wondered what would happen if we used the same gene therapy in a mouse model of obesity induced by high-fat diet (HFD), triggering insulin insensitivity. We demonstrated that expression of IRA induced a decrease in blood glucose to be used by organs/tissues and an amelioration of the hepatic damage due to fat accumulation, being more efficient than IRB expression, as previously described. Therefore, we believe that this kind of gene therapy approach could be beneficial to regulate the important processes involved in the development of non-alcoholic fatty liver disease (NAFLD) associated with obesity.

“Our study is significant to the field of metabolism and, in particular, obesity and NAFLD, highly prevalent conditions worldwide.”

**What are the potential implications of these results for your field of research?**

Our study is significant to the field of metabolism and, in particular, obesity and NAFLD, highly prevalent conditions worldwide. Our findings are very important since there is not yet an efficient treatment for NAFLD. Our gene therapy approach could reduce, or at less delay, the development of this disease, becoming a promising tool in this field.

**What are the main advantages and drawbacks of the model system you have used as it relates to the disease you are investigating?**

As has already been mentioned, obesity is considered the epidemic of the 21st century, and the induction of this condition in mice by a HFD mimics the situation developed in humans. Indeed, these HFD-fed mice are obese and insulin resistant within a few weeks, which is a quick way to study the related pathologies we are talking about.

The main drawback is that in our model we maintained the HFD diet after adeno-associated virus (AAV) administration, which can be different to what occurs in humans, since after diagnosis people are suggested to decrease the fat intake. Therefore, we think that our results could have been even clearer with a reduction of hyperlipidic diet administered after virus injection.

**What has surprised you the most while conducting your research?**

I would say what has surprised me the most was the strong tissue-selective response obtained only for expressing insulin receptor isoforms by injecting a few viruses. I mean that tiny molecules are able to reach liver because of its tropism, finally resulting in an improvement in the damage caused. For instance, there were significant and noticeable improvements in steatosis levels in the experimental groups fed a HFD that were injected with the AAVs.

**Figure:**
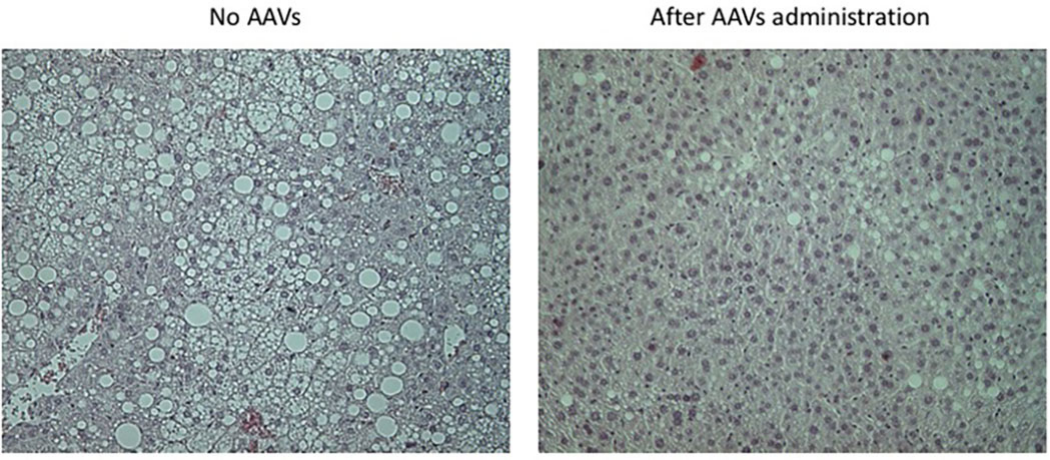
**Significant regression of the degree of steatosis, particularly in response to IRA expression, in HFD-fed mice following administration of AAVs.**

**Describe what you think is the most significant challenge impacting your research at this time and how will this be addressed over the next 10 years?**

One significant challenge that remains in this field is to discover a tool, either drugs or another approach, able to treat liver damage and the related complications. Over the next 10 years, this will be addressed by finding resources compatible with humans and promoting collaboration and communication between research groups and doctors that focus on the same, as well as by fostering translational medicine.

“[…] one of the main concerns of early-career scientists is funding […]”

**What changes do you think could improve the professional lives of early-career scientists?**

From my point of view, one of the main concerns of early-career scientists is funding, especially here in Spain due to the little accessibility and investment. For that reason, there should be an increase in the number of grants by the government in order to have more opportunities to apply for. It is also important to encourage young scientists to go abroad by increasing travel grants to improve their research and foster training courses to complete their CVs. Another change could be by promoting collaborative studies with researchers in the same field to share knowledge and feedback on ideas.

**What's next for you?**

What is next for me personally is that I will be finishing my PhD within the next few years. I would like to continue researching metabolic pathologies, so I hope to obtain a good research position to extend my knowledge.
